# Supplementary material for: Social isolation is associated with microRNA expression changes in the visual system of poison frog tadpoles
Source: bioRxiv. 2026 Sep 11:2026.09.04.749321. Preprint. [Version 1] doi: 10.64898/2026.09.04.749321 (PMC13622530; doi:10.64898/2026.09.04.749321)
Supplement: Supplementary file 1 [file NIHPP2026.09.04.749321V1-supplement-1.pdf]

bioRxiv preprint doi: https://doi.org/10.64898/2026.09.04.749321; this version posted September 11, 2026. The copyright holder for this preprint (which was not certified by peer review) is the author/funder, who has granted bioRxiv a license to display the preprint in perpetuity. It is made available under a CC-BY 4.0 International license.

Supplementary Figures

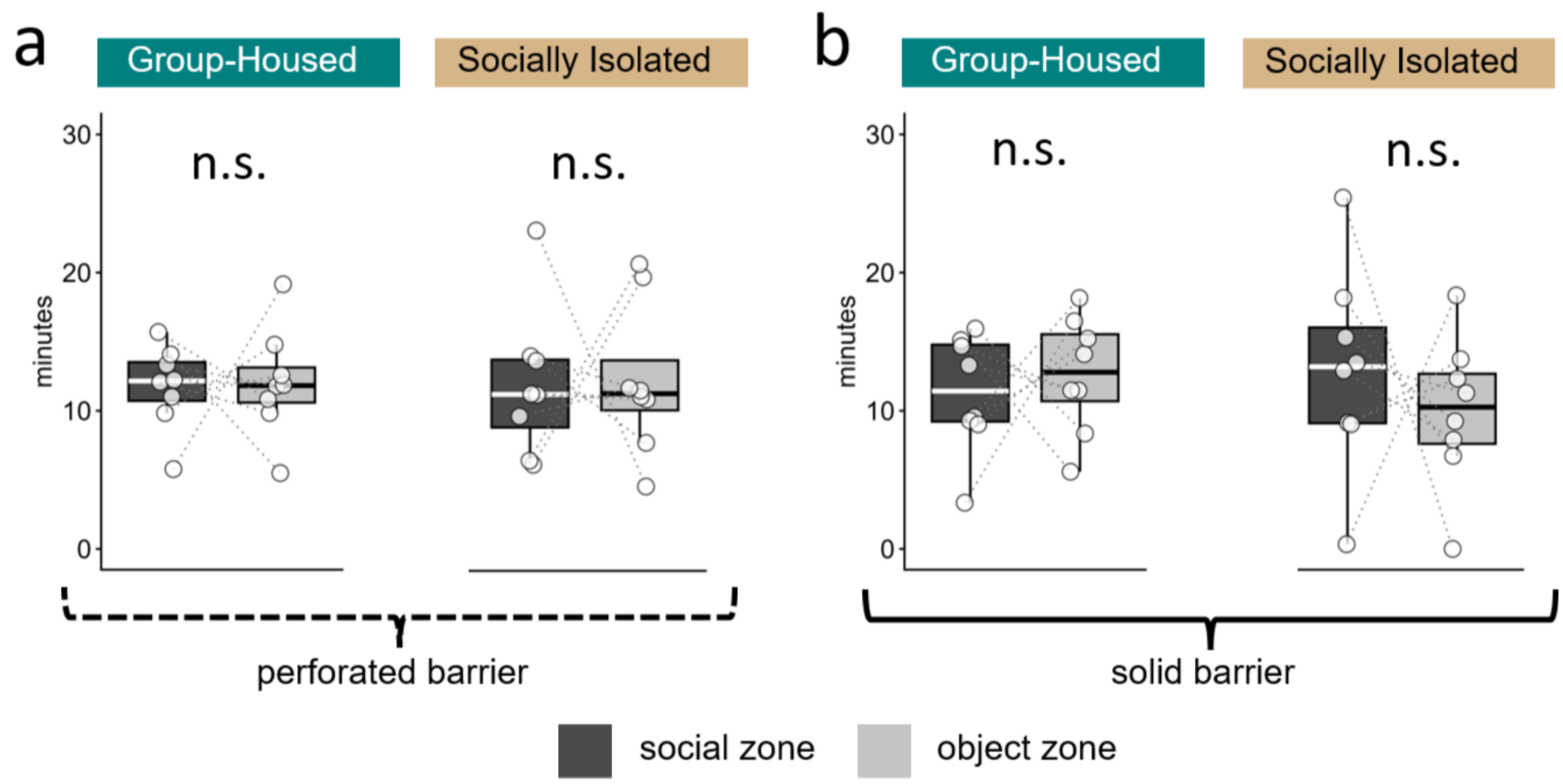

Fig. S1: Tadpoles show no zone preference during the baseline acclimation period. Time in the social (dark gray) and object (light gray) zones during the 30-minute acclimation phase before stimuli introduced. Panels show barrier types separating focal tadpoles from stimuli (a, perforated, permitting chemical/visual/water exchange; b, solid, visual-only access), split by social housing condition (group-housed, socially isolated). Dotted lines connect zone values from the same tadpole. Boxes show median and interquartile range. No group differed between zones. (a) perforated barrier (chem/visual/water access): group-housed ($\chi^2_1 = 0.03$, $p_{adj} = 0.907$), socially isolated ($\chi^2_1 = 0.01$, $p_{adj} = 0.907$). (b) solid barrier (visual-only access): group-housed ($\chi^2_1 = 0.44$, $p_{adj} = 0.771$), socially isolated ($\chi^2_1 = 0.97$, $p_{adj} = 0.771$). Linear mixed model with a random intercept for tadpole; p-values Benjamini-Hochberg corrected across all barrier x rearing x phase comparisons (n = 8 clutches, one group-housed and one isolated tadpole per clutch, per barrier type). n.s., not significant.

bioRxiv preprint doi: https://doi.org/10.64898/2026.09.04.749321; this version posted September 11, 2026. The copyright holder for this preprint (which was not certified by peer review) is the author/funder, who has granted bioRxiv a license to display the preprint in perpetuity. It is made available under a CC-BY 4.0 International license.

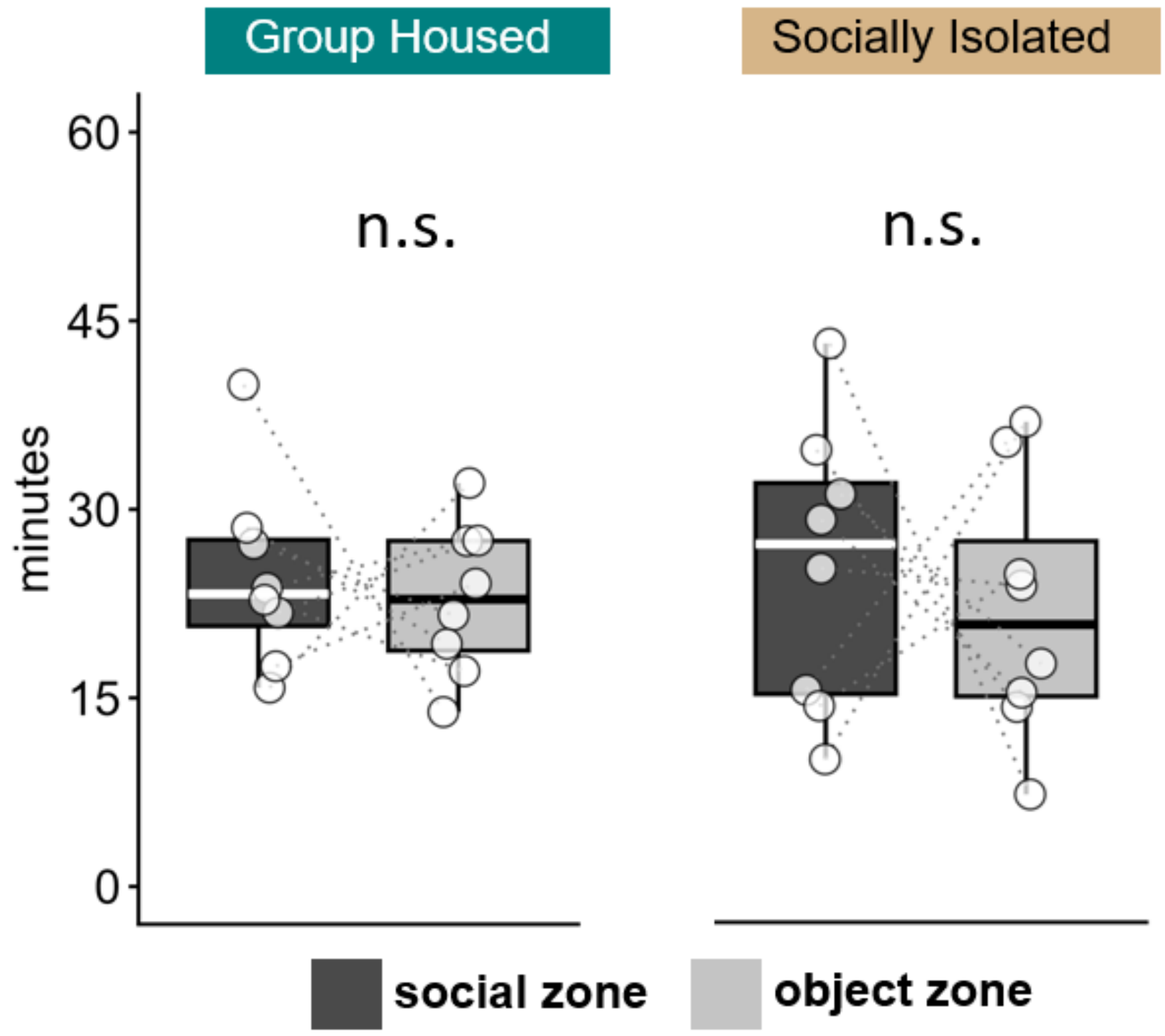

Fig. S2: Time in social place preference zones with visual-only access. Social place preference trials with solid, transparent barriers permitting visual access without water exchange. Time (minutes) in social (dark gray) and object (light gray) zones for group-housed (left) and socially isolated (right) tadpoles. Dotted lines connect occupancy values from the same tadpole. Neither group-housed ($\chi^2_1 = 0.31$, $p_{adj} = 0.771$) nor isolated tadpoles ($\chi^2_1 = 0.46$, $p_{adj} = 0.771$) differed between zones. Separate animals from Fig. 1: one GH and one SI per clutch (n = 8 clutches). Boxes show median and interquartile range.

bioRxiv preprint doi: https://doi.org/10.64898/2026.09.04.749321; this version posted September 11, 2026. The copyright holder for this preprint (which was not certified by peer review) is the author/funder, who has granted bioRxiv a license to display the preprint in perpetuity. It is made available under a CC-BY 4.0 International license.

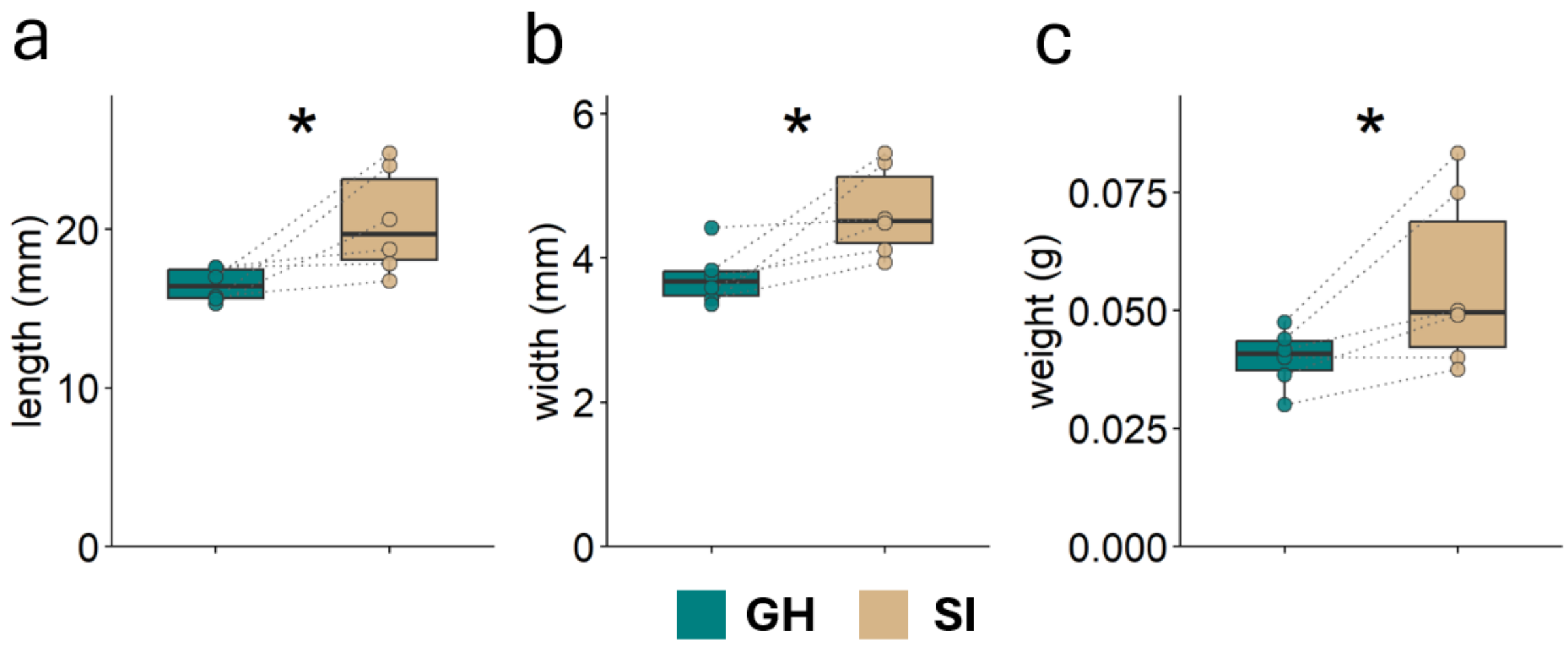

Fig. S3: Socially isolated tadpoles are larger than stage-matched group-housed clutchmates. Size comparison of group-housed (GH, teal) and socially isolated (SI, tan) *A. femoralis* tadpoles. (a) length ($t_5 = 2.65$, $p = 0.046$), (b) width ($t_5 = 3.02$, $p = 0.029$), and (c) weight ($t_5 = 2.73$, $p = 0.041$). Points are clutch/condition means (n = 6 clutches; dotted lines connect paired clutches, * $p < 0.05$).

bioRxiv preprint doi: https://doi.org/10.64898/2026.09.04.749321; this version posted September 11, 2026. The copyright holder for this preprint (which was not certified by peer review) is the author/funder, who has granted bioRxiv a license to display the preprint in perpetuity. It is made available under a CC-BY 4.0 International license.

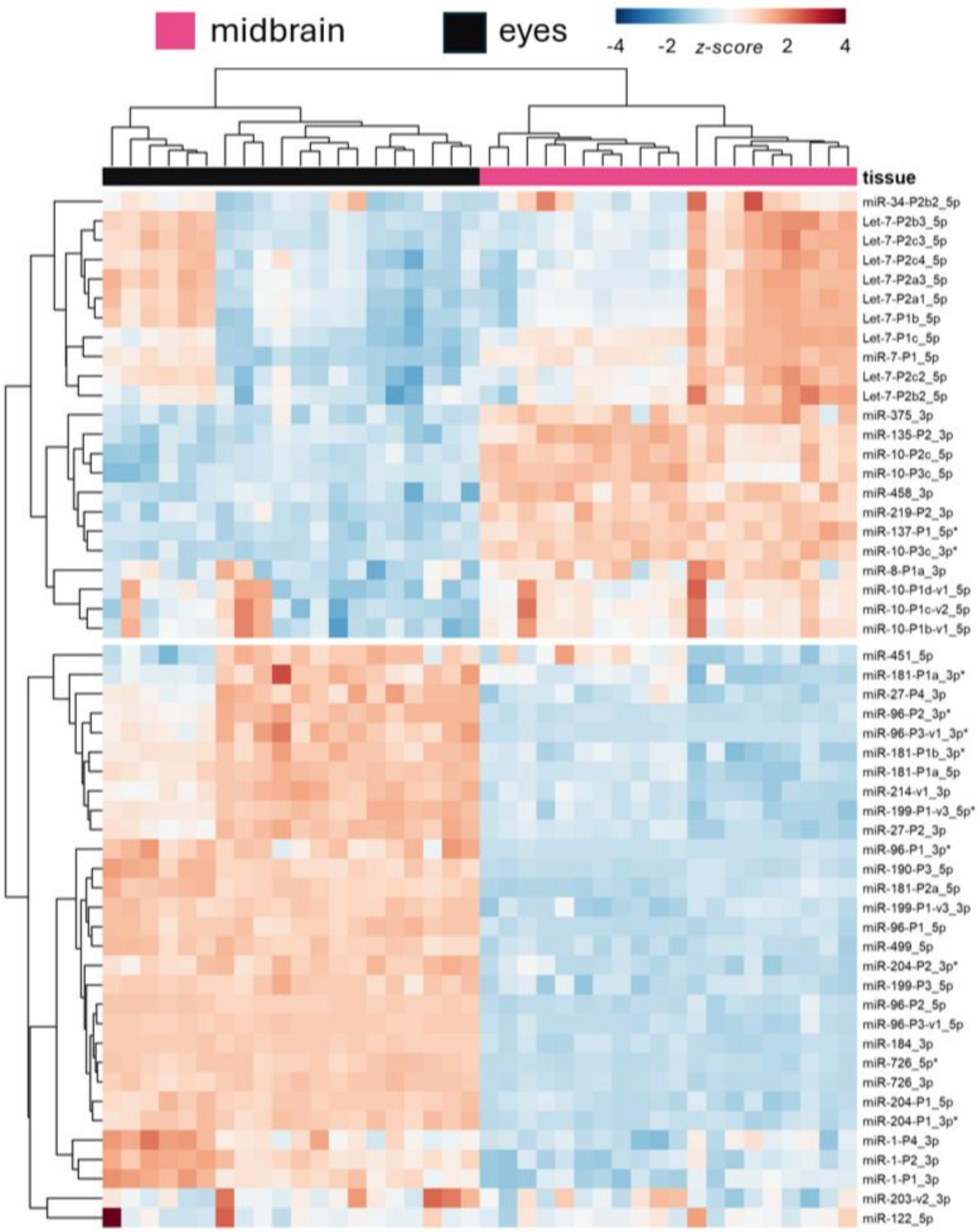

Fig S4: Unsupervised clustering separates libraries by tissue. Heatmap of the top 20% most variable miRNAs (53 miRNAs) across midbrain (n = 20, pink) and eye (n = 20, black) libraries. Values are variance stabilized counts (DESeq2) centered and scaled to unit variance by row (z-score; color bar). miRNA names follow MirGeneDB naming convention; * denotes the non-canonical arm.

bioRxiv preprint doi: https://doi.org/10.64898/2026.09.04.749321; this version posted September 11, 2026. The copyright holder for this preprint (which was not certified by peer review) is the author/funder, who has granted bioRxiv a license to display the preprint in perpetuity. It is made available under a CC-BY 4.0 International license.

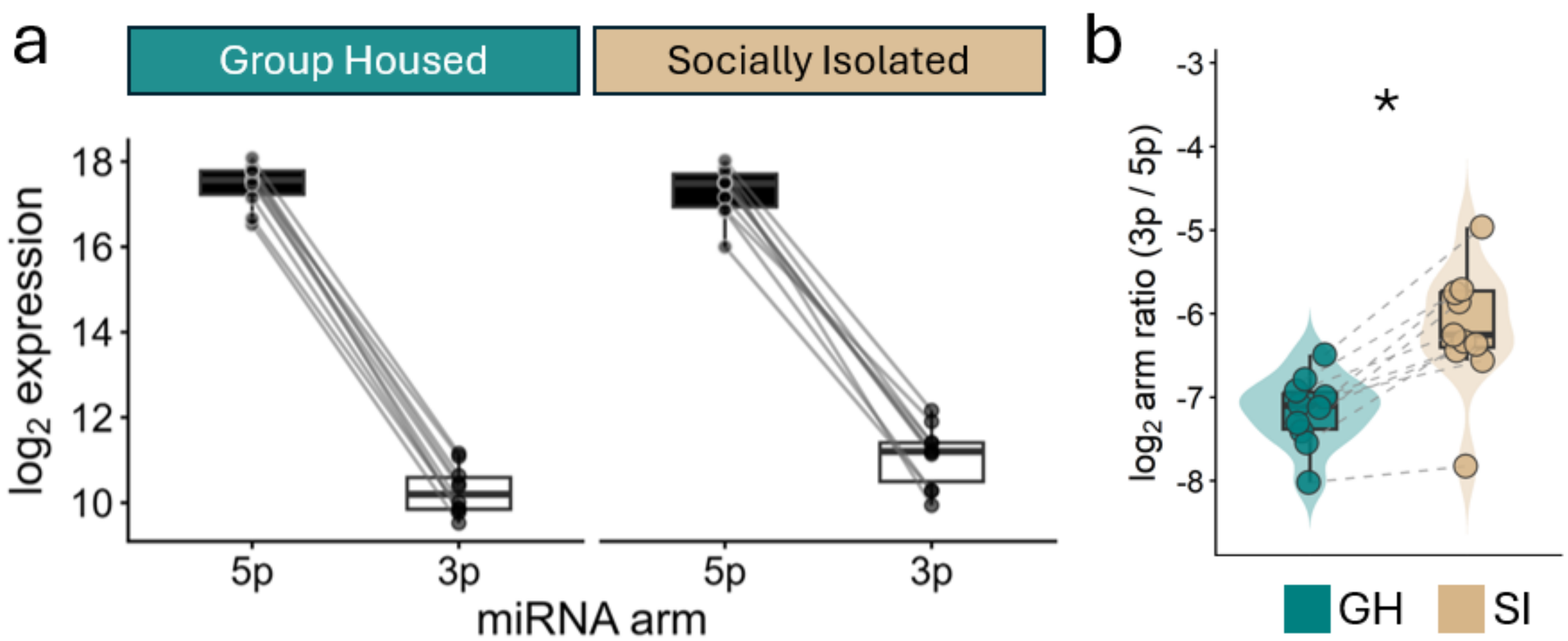

Fig. S5: miR-10-P3b arm dosage shifts in socially isolated midbrains. (a) Paired log_2 expression of miR-10-P3b 5p and 3p arms in group-housed (teal, left) and socially isolated (right, tan) tadpole midbrains. Gray lines connect arms within each midbrain library. The 5p arm was expressed far higher than the 3p arm in both conditions, and this separation slightly narrows in isolated midbrains. (b) Per-library log_2 arm ratio. Negative values indicate higher 5p expression. Unlike miR-124 (Fig. 3) the ratio remains highly 5p dominant across social conditions (linear model, $\beta_{SI} = 0.96$, $p_{adj} = 0.016$; Hedges' g = 1.52, 95% CI [0.63, 3.26]; Cliff's δ = 0.80, [0.38, 1.00]; clutch-paired Wilcoxon signed-rank, n = 10, p = 0.002). * $p_{adj} < 0.05$**.**

bioRxiv preprint doi: https://doi.org/10.64898/2026.09.04.749321; this version posted September 11, 2026. The copyright holder for this preprint (which was not certified by peer review) is the author/funder, who has granted bioRxiv a license to display the preprint in perpetuity. It is made available under a CC-BY 4.0 International license.

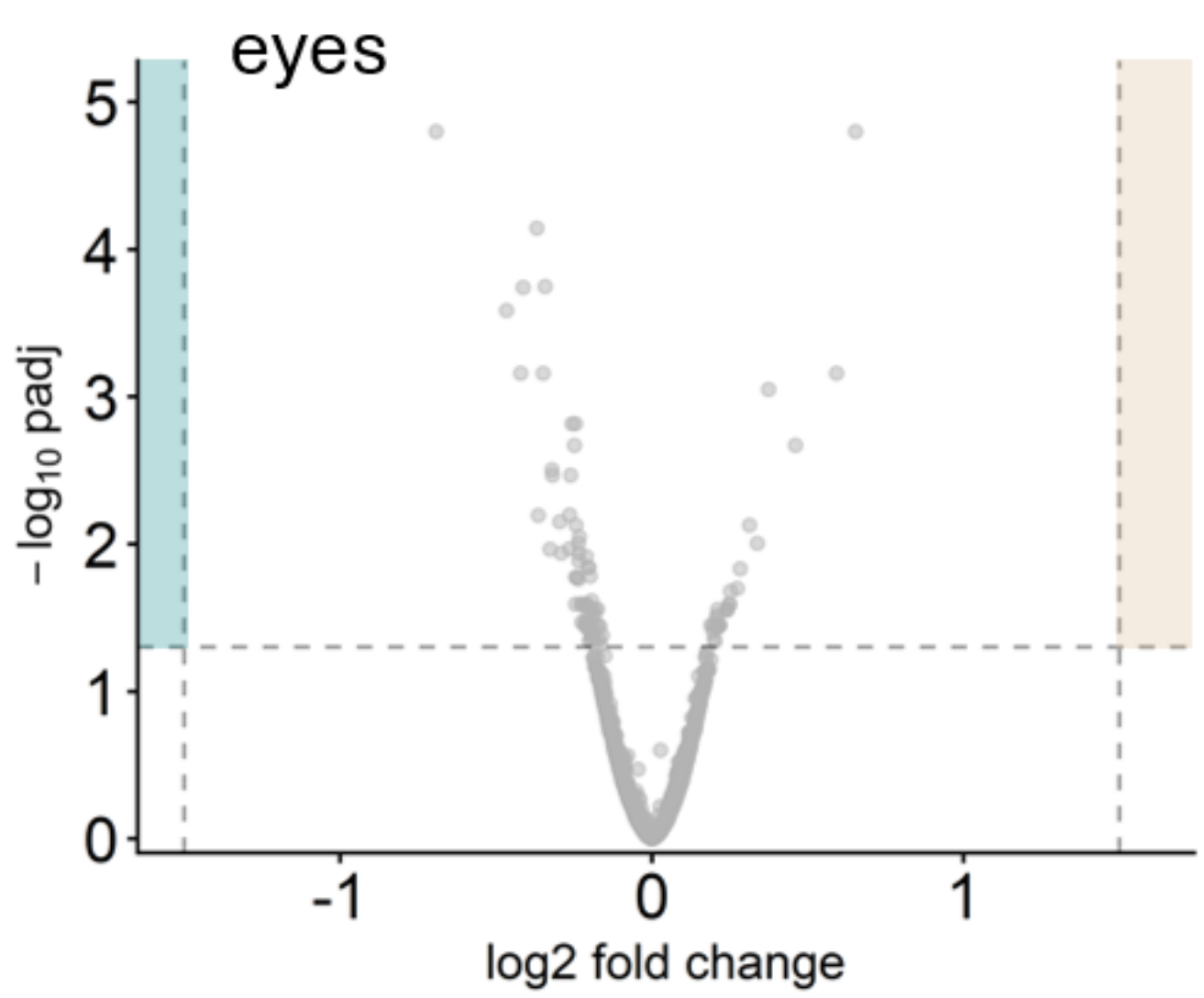

Fig. S6: No transcript is differentially expressed between housing conditions in eyes. Volcano plot of mRNA differential expression between group-housed (GH) and socially isolated (SI) eyes. Dashed lines mark significance and fold-change cutoffs ($p_{adj} < 0.05$, $|log_2FC| > 1.5$).
